# Autophosphorylation of serine 608 in the p85 regulatory subunit of wild type or cancer-associated mutants of phosphoinositide 3-kinase does not affect its lipid kinase activity

**DOI:** 10.1186/1471-2091-13-30

**Published:** 2012-12-27

**Authors:** Meredith J Layton, Mirette Saad, Nicole L Church, Richard B Pearson, Christina A Mitchell, Wayne A Phillips

**Affiliations:** 1 The Department of Biochemistry and Molecular Biology, Monash University, Clayton, VIC, 3800, Australia; 2 Surgical Oncology Research Laboratory, Peter MacCallum Cancer Centre, St Andrew’s Place, East Melbourne, VIC, 3002, Australia; 3 Cancer Signalling Laboratory, Peter MacCallum Cancer Centre, St Andrew’s Place, East Melbourne, VIC, 3002, Australia; 4 Sir Peter MacCallum Department of Oncology, University of Melbourne, Parkville, VIC, 3010, Australia; 5 Department of Surgery, St. Vincent's Hospital, University of Melbourne, Parkville, VIC, 3010, Australia; 6 Department of Biochemistry and Molecular Biology, University of Melbourne, Parkville, VIC, 3010, Australia; 7 The Ludwig Institute for Cancer Research, Royal Melbourne Hospital, PO Box 2008, Parkville, 3050, Australia

**Keywords:** PI3K, PIK3CA, Phosphoinositide, Kinase, Mutation, Oncogene, Phosphorylation

## Abstract

**Background:**

The α-isoform of the Type 1A Phosphoinositide 3-kinases (PI3Kα) has protein kinase activity as well as phosphoinositide lipid kinase activity. The best described substrate for its protein kinase activity is its regulatory subunit, p85α, which becomes phosphorylated on Serine 608. Phosphorylation of Serine 608 has been reported to down-regulate its lipid kinase activity.

**Results:**

We have assessed whether oncogenic mutants of PI3Kα, which have up-regulated lipid kinase activity, have altered levels of Serine 608 phosphorylation compared to wild type PI3Kα, and whether differential phosphorylation of Serine 608 contributes to increased activity of oncogenic forms of PI3Kα with point mutations in the helical or the kinase domains. Despite markedly increased lipid kinase activity, protein kinase activity was not altered in oncogenic compared to wild type forms of PI3Kα. By manipulating levels of phosphorylation of Serine 608 *in vitro*, we found no evidence that the protein kinase activity of PI3Kα affects its phosphoinositide lipid kinase activity in either wild-type or oncogenic mutants of PI3Kα.

**Conclusions:**

Phosphorylation of p85α S608 is not a significant regulator of wild-type or oncogenic PI3Kα lipid kinase activity.

## Background

Phosphoinositide 3-kinases (PI3Ks) are a ubiquitous family of lipid kinases that catalyse the phosphorylation of phosphoinositide lipids at the 3’ position on the inositide ring [1]. The Class 1A sub-group exists as heterodimers of a p110 catalytic subunit and a p85 regulatory subunit that can phosphorylate three phosphoinositides (PI, PI-(4)-P and PI-(4,5)-P_2_) *in vitro*, but are thought to primarily phosphorylate PI-(4,5)-P_2_*in vivo* to form PI-(3,4,5)-P_3_ (PIP_3_) [1]. PIP_3_ resides almost exclusively in the plasma membrane and propagates signals by recruiting and activating a variety of downstream proteins that contain PIP_3_ binding domains (primarily Pleckstrin Homology (PH) domains) [2]. PI3K/PIP_3_ signalling regulates a wide range of cellular processes including cell growth, survival, glucose metabolism and migration [1,3].

The prototypic Class 1A PI3K is a heterodimer of the α isoforms of p110 and p85 (p110α/p85α or PI3Kα). Mutations in *PIK3CA*, the gene encoding p110α, are oncogenic [4-7] and are frequently reported in breast and colon cancers, where they are found in around 25% of human tumours [8-11]. Tumour-associated *PIK3CA* mutations are all somatic, mono-allelic single base changes that result in single amino acid substitutions. The majority (>80%) of mutations cluster in exon 9 (which codes for the helical domain) or exon 20 (which codes for the kinase domain), most commonly E542K and E545K in exon 9 and H1047R in exon 20 [4,12]. Cancer-associated, mutated forms of PI3Kα are associated with increased phosphoinositide kinase activity [4-6,13], leading to up-regulation of downstream signalling events such as phosphorylation of Akt and S6 [5,14].

Class 1A PI3Ks also have protein kinase activity. The p110α catalytic subunit can phosphorylate its regulatory subunit, p85α, at Serine 608 (S608). Phosphorylation of this site has been reported to result in feed-back inhi-bition by down-modulating the lipid kinase activity of p110α [15-17], however the role of phosphorylation of S608 in signalling by endogenous PI3Kα and the structural mechanism of down-modulation of lipid kinase activity by S608 phosphorylation are not well described. We have assessed the possibility that the increased lipid kinase activities of oncogenic mutants of PI3Kα could be partly due to alterations in the phosphorylation of S608. Rather than make point mutations in S608, which can potentially subtly alter the structure and thus the activity of p110α/p85α heterodimers, we have manipulated the levels of phosphorylation of S608 of highly-purified, recombinant p110α/p85α *in vitro* and tested the effect on lipid kinase activity. Neither complete dephosphorylation nor a high percentage occupancy of S608 by a phosphate group significantly altered the lipid kinase activity of wild-type PI3Kα. The levels and kinetics of S608 phosphorylation in two oncogenic mutants, E545K and H1047R, were not significantly different to that of wild-type p110α/p85α and the lipid kinase activities of mutant PI3Kα were similarly unaffected by phosphorylation of S608. This suggests that phosphorylation of S608 is not a significant regulator of PI3Kα lipid kinase activity.

## Results

### Expression, purification and characterisation of recombinant wild-type and mutant PI3Kα

**Figure 1:**
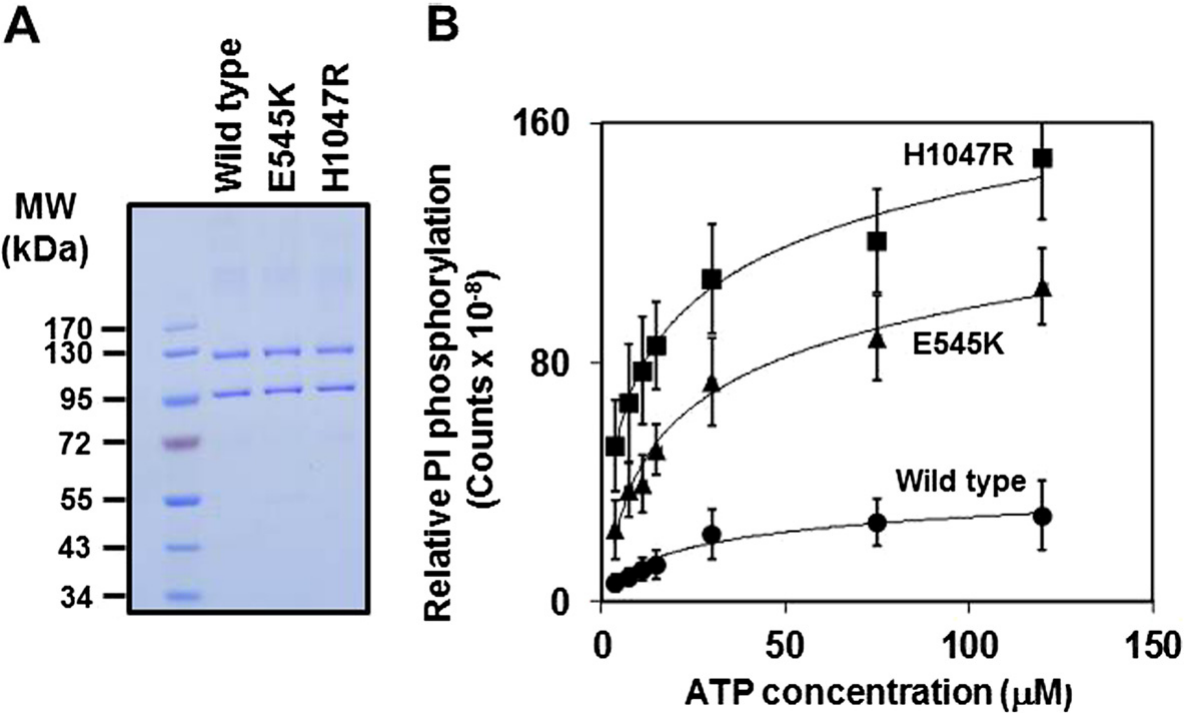
**Characterisation of purified**, **recombinant PI3Ks.A**. SDS-PAGE and Coomassie Blue staining of molecular weight standards (Fermentas, Lane 1), 0.5 μg purified p110αEE^WT^/p85α (lane 2), 0.5 μg purified p110αEE^E545K^/p85α (lane 3) and 0.5 μg purified p110αEE^H1047R^/p85α (lane4). **B**. Purified, recombinant PI3Ks were assayed for lipid kinase activity with increasing concentrations of ATP using PI-(4,5)-P_2_ as a substrate. Reactions were stopped after 20 minutes using 1 M HCl. The amount of ^32^P-PI-(3,4,5)-P_3_ generated was quantified using a phosphorimager and displayed as mean ± SEM, n = 4.

The same strategy that was used to express and purify active, recombinant bovine p110α/p85α [18] was used to generate human, C-terminally EE-tagged, full length, wild-type p110α/p85α in Sf9 insect cells using the Bac-N-Blue baculovirus system. When Sf9 cells were co-infected with baculoviruses encoding both subunits, p85 was expressed at higher levels than p110αEE, therefore to obtain purified p110αEE/p85α, affinity chromatography using an antibody directed against the epitope tag (EE mAb) was used to capture the enzyme complex but not excess p85α. Competitive elution with an EE-tag peptide (EYMPME), followed by anion exchange chromatography were performed as described [18]. PI3K purified in this way comprises a homogeneous 1:1 complex of the p110α and p85α subunits as assessed by both SDS-PAGE (Figure 1A) and analytical size exclusion chromatography [18]. Recombinant p110αEE/p85α was estimated to be > 95% pure based on densitometry of the relative levels of all Coomassie blue-stained bands in each lane and no peptides other than those derived from p110αEE or p85α were detected by LC-MS/MS of tryptic digests of the purified recombinant complex. We have also expressed and purified to homogeneity EE-tagged complexes of p85α with p110α containing two of the most common cancer-associated mutations from two different regions of p110α (E545K in exon 9 and H1047R in exon 20) (Figure 1A).

Oncogenic mutants of PI3Kα have been reported to have higher lipid kinase activity than wild-type [4-6,13]. As shown previously [15,19], recombinant forms of the p110αEE^E545K^/p85α and p110αEE^H1047R^/p85α complexes have higher lipid kinase activities than p110αEE^WT^/p85α. The H1047R mutant had higher activity than the E545K mutant (Figure 1B). Kinetic parameters were derived for recombinant wild type and mutant p110αEE/p85α complexes by varying the concentration of ATP and measuring initial reaction rates (Table 1), and showed that, when PI was used as the substrate, the K_m_ for H1047R and E545K were similar to wild type. When PI-(4,5)-P_2_ was used as a substrate, the K_m_ for H1047R was decreased approximately 2-fold compared to wild type, but that for E545K was not significantly different. Both mutants showed a significant 3 to 4-fold increase in V_max_ when either PI or PI-(4,5)-P_2_ was used as a substrate, with the V_max_ for H1047R being significantly higher than that for E545K (Table 1). The kinetic characteristics of purified, recombinant, EE-tagged forms of wild-type and mutant PI3Kα are therefore essentially the same as those previously reported for purified, recombinant His-tagged forms of PI3Kα [15,19] and endogenous wild-type and mutant PI3K [4,5].

**Table 1:** **Kinetic analysis of *in vitro *lipid kinase activity of p110**α**EE**^**WT**^/**p85**α, **p110**α**EE**^**E545K**^/**p85**α **and p110**α**EE**^**H1047R**^/**p85**α

		**Wild type**	**E545K**	**H1047R**
**PI kinase activity**^**a**^	**V**_**max**_^**b**^	35 ± 13^c^	118 ± 12	150 ± 13
	**K**_**m**_ (**μM**)	23 ± 3	22 ± 6	14 ± 7
**PI**-(**4**,**5**)-**P**_**2**_**kinase activity**	**V**_**max**_	35 ± 8	117 ± 13	146 ± 15
	**K**_**m**_ (**μM**)	22 ± 5	19 ± 6	9 ± 3

^a^PI or PI-(4,5)-P_2_ were used as substrates to generate PI-3-P or PI-(3,4,5)-P_3_.

^b^Michaelis-Menten kinetic parameters (substrate affinity (K_m_) and maximum reaction velocity (V_max_)) for the production of PI-3-P or PI-(3,4,5)-P_3_ were calculated from assays in which the concentration of ATP was varied.

^c^K_m_ and V_max_ are shown as mean ± SEM, n = 4.

### Effect of dephosphorylation of p85α S608 on PI3Kα lipid kinase activity

S608 in the p85 subunit is a substrate for the protein kinase activity of PI3Kα [17, 20]. Phosphorylation of S608 by p110 is reported to down-regulate PI3Kα lipid kinase activity [17,20]. This raises the possibility that higher lipid kinase activities of oncogenic mutants of PI3Kα could be partly due to alterations in the levels of phosphorylation of S608.

**Figure 2:**
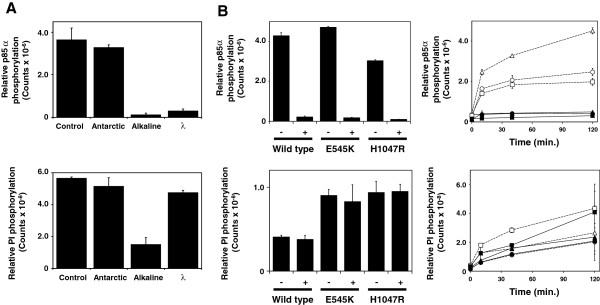
**Dephosphorylation of p85**α **S608 does not alter PI kinase activity.A**. Dephosphorylation of PI3Kα using different protein phosphatases. Purified, recombinant p110αEE^WT^/p85α (200 ng) was assayed for protein (upper panel) or PI (lower panel) kinase activity in the presence of no phosphatase (control), 2 U Antarctic Phosphatase, 10 U Calf Intestinal Alkaline Phosphatase or 40 U Lambda (λ) Protein Phosphatase. Reactions were stopped after 30 minutes using 1 M HCl. **B**. Lipid kinase activity of dephosphorylated wild-type and mutant PI3Kα. 200 ng purified, recombinant p110αEE^WT^/p85α, p110αEE^E545K^/p85α or p110αEE^H1047R^/p85α was assayed for protein kinase activity (upper panel) or lipid kinase activity using PI as a substrate (lower panel) in the presence or absence of 40 U λ Protein Phosphatase. Reactions were stopped after 40 minutes using 1 M HCl. **C**. Time courses of phosphorylation of p85α S608 and PI in the presence or absence of a protein phosphatase. 200 ng purified, recombinant p110αEE^WT^/p85α (circles), p110αEE^E545K^/p85α (triangles) or p110αEE^H1047R^/p85α (squares) was assayed for protein kinase activity (upper panel) or lipid kinase activity using PI as a substrate (lower panel) in the presence (closed symbols) or absence (open symbols) of 20 U λ Protein Phosphatase. Reactions were stopped after the indicated times using 1 M HCl.

To better compare lipid kinase activities of wild-type and mutant PI3Kα that were phosphorylated or unphosphorylated at S608, we opted to manipulate phosphorylation levels of recombinant p110αEE/p85α complexes *in vitro* rather than to make point mutations in S608. To perform lipid kinase assays with dephosphorylated PI3Kα, it was necessary to find a phosphatase that dephosphorylated p85α but not inositol lipids. We compared three different protein phosphatases (Figure 2A). We have previously used Antarctic Phosphatase to dephosphorylate affinity-precipitated proteins for characterisation on 2D gels [21], however Antarctic Phosphatase was inactive at temperatures >4°C that are required for significant lipid and protein kinase activity of PI3Kα, and so had no effect on levels of phosphorylation of p85α or PI at room temperature (Figure 2A). Alkaline phosphatase is a broad spectrum phosphatase that was able to dephosphorylate both S608 and PI-3-P. In contrast, recombinant λ phosphatase dephosphorylated S608 but not PI-3-P (Figure 2A). λ phosphatase was therefore added to PI kinase assays to continually remove phosphate groups from S608 and allow comparison of the lipid kinase activity of phosphorylated and dephosphorylated PI3Kα.

As expected, inclusion of λ phosphatase in kinase assays resulted in no significant incorporation of ^32^P-labelled phosphate groups into p85α, demonstrating that levels of p85α phosphorylation were low in the presence of λ phosphatase (Figure 2B, upper panel). Dephosphorylated p110αEE^WT^/p85α did not have a significantly different PI kinase activity to p110αEE^WT^/p85α that became phosphorylated on p85α over the course of the assay (Figure 2B, lower panel). Similarly, the E545K and H1047R mutants did not have significantly different PI kinase activities when p85α was either dephosphorylated or phosphorylated, although the PI kinase activities of p110αEE^E545K^/p85α and p110αEE^H1047R^/p85α were higher than that of p110αEE^WT^/p85α as expected (Figure 2B, lower panel). Interestingly, as observed previously [15], the protein kinase activities of p110αEE^E545K^/p85α and p110αEE^H1047R^/p85α were not significantly different to that of p110αEE^WT^/p85α (Figure 2B, upper panel).

It is possible that the stoichiometry of phosphorylation of S608 remains low after the 40 minute kinase reaction used, and thus lipid kinase activities of dephosphorylated and only partially phosphorylated p110αEE/p85α were compared in Figure 2B. We therefore performed a time-course to determine whether a difference in activity of phosphorylated and dephosphorylated p110αEE/p85α became apparent with increasing levels of phosphorylation of p85α. Levels of phosphorylation of S608 became saturated after approximately 30 minutes (Figure 2C, upper panel). In some assays, the level of phosphorylation of the E545K mutant was apparently higher than wild-type PI3Kα or the H1047R mutant, although this was an inconsistent observation and was not significantly different in most assays. When λ phosphatase was added, lack of incorporation of ^32^P into p85α demonstrated that λ phosphatase continued to dephosphorylate S608 throughout the assay. (Figure 2C, lower panel, closed symbols). Despite apparently saturating phosphorylation of S608 between 40 and 120 minutes, the difference in the PI kinase activity of phosphorylated and dephosphorylated wild-type, E545K or H1047R PI3Kα did not increase over time (Figure 2C, lower panel), suggesting that phosphorylation of S608 does not regulate the PI kinase activity in either wild-type or mutant PI3Kα.

### Effect of phosphorylation of p85α S608 on PI3Kα lipid kinase activity

**Figure 3:**
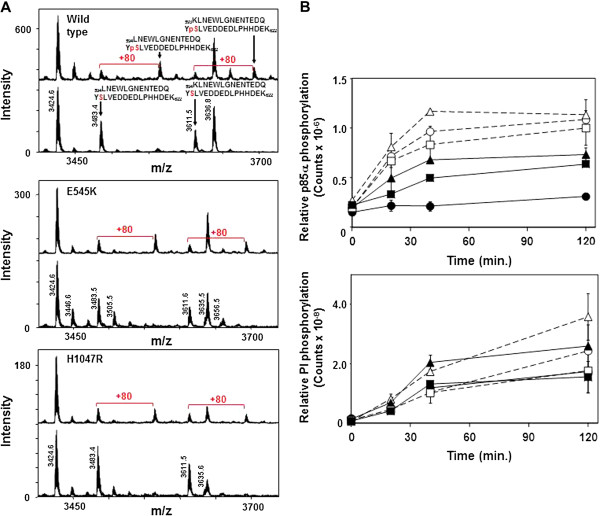
**Phosphorylation of p85**α **S608 does not alter PI kinase activity.** 25 μg purified, recombinant p110αEE^WT^/p85α, p110αEE^E545K^/p85α or p110αEE^H1047R^/p85α was incubated with (phosphorylated) or without (mock) 1 mM ATP for 16 hr. Excess ATP was removed by buffer exchange into TBS containing 10 mM 2-mercaptoethanol then phosphorylated or mock phosphorylated PI3Kα was concentrated to approximately 0.5 mg/ml. **A**. MALDI-MS spectrum from m/z 3400–3750 of a Fe^3+^ IMAC-enriched tryptic digest from phosphorylated (upper spectrum) or mock phosphorylated (lower spectrum) p110αEE^WT^/p85α, p110αEE^E545K^/p85α and p110αEE^H1047R^/p85α. The intensity scale for these spectra were normalised to the peak at m/z 3424.5, which corresponds to a peptide encompassing residues 35–66 from p85α (_35_GSLVALGFSDGQEAKPEEIGWLNGYNETTGER_66_) that is not phosphorylated by the protein kinase activity of p110αEE/p85α. Serine 608 from p85α was observed within 2 overlapping peptides of m/z 3483.4 corresponding to residues 594–622 from p85α (_594_LNEWLGNENTEDQYSLVEDDEDLPHHDEK_662_) and m/z 3611.5 corresponding to residues 593–622 from p85α (_593_KLNEWLGNENTEDQYSLVEDDEDLPHHDEK_662_). Incubation with ATP resulted in the appearance of peptides of masses m/z 3563.5 and m/z 3691.6 which are 80 mass units (the mass of a phosphoryl- group) greater than m/z 3483.4 and m/z 3611.5. **B**. 250 ng of phosphorylated (closed symbols) or mock phosphorylated (open symbols) p110αEE^WT^/p85α (circles), p110αEE^E545K^/p85α (triangles) or p110αEE^H1047R^/p85α (squares) were assayed for PI (lower panel) or protein (upper panel) kinase activities as described above.

To ensure that the apparent lack of difference in PI kinase activity of phosphorylated and dephosphorylated PI3Kα was not due to a low stoichiometry of phosphorylation, even when levels were apparently saturated, we allowed the phosphorylation reaction to proceed for 16–24 hours using unlabelled ATP or no ATP for the mock phosphorylation reaction. Phosphorylated and mock-phosphorylated PI3Kα were buffer exchanged to remove excess cold ATP, then the extent of phosphorylation of S608 was measured using MALDI-MS of tryptic digests of wild-type and mutant PI3Kα. Overlapping tryptic peptides containing S608 were observed at m/z 3483.4 and m/z 3611.5 in mock or unphosphorylated PI3Kα (Figure 3A, lower spectrum in each panel). Phosphorylation of PI3Kα led to the appearance of peptides at m/z 3563.5 and m/z 3691.6 (Figure 3A, upper spectrum in each panel), which are 80 mass units greater than the masses of the peptides containing non-phosphorylated S608 and suggest that these peptides incorporated one phosphate group per peptide. There was no evidence for phosphorylation of peptides other than those containing S608. To estimate the extent of phosphorylation of S608, we normalised the spectra to the height of a peptide at m/z 3426.6 that is not altered when PI3Kα is phosphorylated, and compared the heights of the peaks of non-phosphorylated S608-containing peptides. Phosphorylation of p110αEE^WT^/p85α led to almost complete disappearance of the peaks at m/z 3483.4 and m/z 3611.5 corresponding to non-phosphorylated S608, suggesting that at least 90% of p110αEE^WT^/p85α was phosphorylated on S608 (Figure 3A, upper spectrum of panel 1). In support of this, the amount of ^32^P incorporated into p85α in a subsequent protein kinase time-course assay was negligible, suggesting that nearly all S608 residues were already occupied by an unlabelled phosphate group (Figure 3B, upper panel). The extent of phosphorylation of p85α S608 in p110αEE^E545K^/p85α and p110αEE^H1047R^/p85α was less than that observed for p110αEE^WT^/p85α, as the heights of the peaks at m/z 3483.4 and m/z 3611.5 were less decreased by phosphorylation compared to the height of the same peaks in mock-phosphorylated p110αEE^E545K^/p85α and p110αEE^H1047R^/p85α (Figure 3A, panels 2 & 3), suggesting that a small proportion of mutant PI3Kα remained unphosphorylated after 16 hours. In agreement, low levels of ^32^P were incorporated into p85α in subsequent protein kinase time-course assays (Figure 3B, upper panel), suggesting that a small proportion of S608 in the E545K and H1047R mutants was not occupied by an unlabelled phosphate group. Nevertheless, the majority of p110αEE^E545K^/p85α and p110αEE^H1047R^/p85α was phosphorylated on S608 under these conditions.

The PI kinase activities of phosphorylated and mock-phosphorylated wild-type PI3Kα were not significantly different over a 2 hour time-course. Similarly, the PI kinase activities of phosphorylated and mock-phosphorylated E545K and H1047R PI3Kα were not significantly different, although the activities of H1047R, and to a lesser extent E545K, were higher than that of wild-type PI3Kα. Nearly stoichiometric phosphorylation of S608 therefore did not affect the PI kinase activity of wild-type or mutant PI3Kα, further suggesting that phosphorylation of S608 does not regulate PI kinase activity.

## Discussion

As reported previously for bovine PI3Kα [18], we have successfully expressed and purified recombinant complexes of full-length human p110α and p85α, the prototypic form of Class 1A PI3K using the strategy of selecting for the of p110αEE/p85α complex by placing a 6 amino acid epitope tag at the C-terminus of the p110α, the subunit with limiting expression levels. The addition of C-terminal EE tag to p110α did not appear to affect PI3Kα, as recombinant p110αEE/p85α had high stability and the expected activity and substrate specificity. High purity recombinant PI3Kα allowed us to assess the relative phosphoinositide lipid and protein kinase activities of wild-type and two common, tumour-associated mutants of PI3Kα.

Purified, recombinant oncogenic mutant forms of PI3Kα had higher lipid kinase activities compared to wild-type PI3Kα (Figure 1B) as expected [4-6,13]. The ATP K_m_ was not significantly different between wild-type and mutant forms of PI3Kα (Table 1) as previously reported [19]. However, there was a significant difference in ATP V_max_ when either PI or PI-(4,5)-P_2_ was used as a substrate, in agreement with a previous study [15]. This suggests that mutant forms of PI3Kα phosphorylate and turn over inositol lipids more rapidly, rather than having inherent higher substrate affinities, thus generating increased levels of PIP_3_ and potentially partially explaining increased downstream signalling when the PI3K pathway is activated.

PI3Kα also has protein kinase activity and has been reported to phosphorylate a number of protein substrates [22,23] as well as its own p85α regulatory subunit on S608 [16,17,20]. The role of S608 phosphorylation in the regulation of PI3Kα lipid kinase activity remains controversial. Despite previous reports that phosphorylation of S608 down-regulates the lipid kinase activity of the p110α catalytic subunit, we did not find any evidence to support this by *in vitro* manipulation of the levels of phosphorylation of S608 of purified, recombinant p110αEE/p85α using either a protein phosphatase or saturating pre-phosphorylation. The reason for this discrepancy is not clear. In two studies, semi-purified recombinant PI3Kα was used to show phosphorylation of S608 reduces the lipid kinase activity of p110α [17,20]. Semi-purified PI3Kα can be unstable, thus it is possible that the apparent decrease in the lipid kinase activity of semi-purified PI3Kα over time was due to p110α degradation rather than S608 phosphorylation. A number of other studies have not observed a decrease in lipid kinase activity of recombinant, purified or immunoprecipitated PI3Kα in time-course assays [24-27].

The time-course of PIP_3_ formation by endogenous PI3K in cells shows an initial increase, followed by a decrease in PIP_3_ levels [28,29], but this is largely due to the action of inositol 3- and 5-phosphatases that directly dephosphorylate PIP_3_ to form PI-(4,5)-P_2_ or PI-(3.4)-P_2_ respectively [30,31]. The phosphorylation of S608 in cells increases upon stimulation with insulin or PDGF [16], which mirrors activation of the lipid kinase activity of PI3Kα, thus it is not clear whether phosphorylation of S608 down-regulates endogenous PI3Kα activity. Mutation of S608 to a non-phosphorylatable residue has been shown to decrease lipid kinase activity rather than increase it, as would be expected if mutation to alanine prevented phospho-S608-induced down regulation of PI kinase activity [16], suggesting that mutation of this residue, which resides in the interSH2 domain of p85α, affects PI3Kα lipid kinase activity by altering the structure of PI3Kα rather than by affecting the phosphorylation status.

Phosphorylation of S608 has also been reported to lead to dissociation of the p85α and p110α subunits [16]. Free p110α has higher activity than p110α in complex with p85α but is very unstable and is quickly degraded [32], thus it is possible that the decrease in lipid kinase activity when p85α S608 is phosphorylated could be explained by dissociation of p110α and p85α and rapid degradation of free p110α. However, our highly purified recombinant forms of PI3Kα, when phosphorylated to saturation on p85α S608, showed no evidence of subunit dissociation by size-exclusion chromatography and no evidence of p110α degradation by SDS-PAGE (data not shown). The lack of observed dissociation, degradation or decrease in lipid kinase activity concomitant with increasing p85α S608 phosphorylation in our highly purified, recombinant PI3Kα suggests that previous reports of S608 phosphorylation resulting in down-regulation of lipid kinase activity were due to the use semi-purified recombinant PI3Kα which is non-specifically degraded over time.

A number of mechanisms that regulate PI3Kα enzymatic activity have been described, but no single event has been demonstrated to result in full activation of this enzyme. It is likely that full activation of PI3Kα is a multi-step process. Within the p110α/p85α complex, p110α is both inhibited and structurally stabilised by tight binding to the p85α subunit [32]. Activation of the PI3Kα involves protein-protein interactions that relieve the inhibition of the p110α kinase activity that is due to inter-subunit interactions of p110α with the N-terminal SH2 and inter-SH2 domains of p85α [33]. One mechanism of activation of PI3Kα is binding to specific phosphotyrosine-containing motifs (pYXXM) present in receptor tyrosine kinases (RTKs) and cytoplasmic signalling proteins, such as IRS-1, to the p85α N- and C-terminal SH2 domains [34-36], which disrupts the inhibitory contact between the p85α N-terminal SH2 and the p110α catalytic domain [37,38]. The E545K mutation is thought to increase activity by disrupting the p85α N-terminal SH2/p110α interface, similar to binding of RTKs [37,38]. Binding of a range of other intracellular proteins, such as activated Ras, SH3 domain-containing proteins and small GTPases, have also been reported to activate PI3Kα [39-41] but it is not clear whether binding of these ligands directly activates enzyme activity or whether binding results in activation by translocating PI3Kα to the plasma membrane, where its lipid substrate is located [42,43]. Tyrosine phosphorylation of p85α Y688 by Src family tyrosine kinases has also been shown to increase PI3Kα activity [44], but the mechanism is not known.

Phosphorylation of p85α S652 by PKC [45] (as well as p85α S608 by p110α) has been reported to decrease PI3Kα activity. S608 is in the inter-SH2 domain of p85α, but is not within the section that has been observed to contact p110α [37,38,46], therefore it is not clear whether phosphorylation of S608 could influence the inter-subunit interactions of p110α and p85α. A model of the structure of p110 with the N- terminal SH2, C- terminal SH2 and inter-SH2 domains of p85 [47] suggests that the C-terminal part of the inter-SH2 domain (containing S608) and the C-terminal SH2 domain has the potential to contact the catalytic domain of p110. Activation of PI3Kα due to binding of the p85α C-terminal SH2 domain to phosphotyrosine-containing proteins would be expected to be due to a conformational change that disrupts the interface between the p85α C-terminal SH2 domain and the p110α catalytic domain, analogous to the disruption of the inhibitory contact between the p85α N-terminal SH2 and the p110α catalytic domain. In contrast, for phosphorylation of S608 to decrease PI3Kα enzymatic activity, it would have to stabilise this interface and oppose the phosphotyrosine binding-induced disruption. Regulation of PI3Kα by stabilisation of inter-subunit interactions has not yet been demonstrated.

## Conclusions

Studies showing a decrease in PI kinase activity of wild-type PI3Kα that correlated with increasing p85α S608 phosphorylation suggested the possibility that decreased S608 phosphorylation of oncogenic PI3Kα could be a mechanism underlying its increased PI kinase activity. This study, in which highly purified, recombinant wild-type and oncogenic, mutant PI3Kα was dephosphorylated or fully phosphorylated on serine 608 *in vitro* (thus avoiding point mutations which can potentially alter protein structure and activity), found no evidence that phosphorylation of S608 influenced PI kinase activity. In addition, levels of phosphorylation of oncogenic PI3Kα were not different to that of wild-type PI3Kα.

## Methods

### Production of recombinant proteins

cDNAs encoding full-length human p85α (PIK3R1_HUMAN, [Uniprot: P27986], aa1-724) or full-length human wild-type or mutant (E545K or H1047R) p110α (PIK3CA_HUMAN, [Uniprot: P42336], aa1-1068) with an additional C-terminal spacer (PGG) and a Glu- or EE-epitope tag (EFMPME) [18,48] were subcloned into the transfer plasmid pBlueBac4. Recombinant baculoviruses were produced by co-transfection of these transfer plasmids into Sf9 insect cells with Bac-N-Blue baculovirus DNA (Invitrogen), then plaque purified and amplified as described [49]. EE-epitope tagged p110α (p110αEE) and p85α were co-expressed in exponentially growing Sf9 cells (density = 1.5-2 × 10^6^ cells/ml) by infection with recombinant baculoviruses at a multiplicity of infection (MOI) of between 1 and 10. Wild-type or mutant p110αEE/p85α complexes were purified to homogeneity by anti-EE tag affinity chromatography and anion exchange chromatography as previously described [18,50]. The concentrations of purified, recombinant PI3Ks were quantified by UV spectroscopy using a molar extinction coefficient of 264115 M^-1^ cm^-1^.

### Gel electrophoresis

Recombinant p110αEE/p85α complexes were separated by SDS-PAGE using 10% Tris-glycine gels. Fast Coomassie Blue staining and destaining was carried out using a microwave oven as described [51].

### Phosphoinositide 3-kinase assays

PI3K assays were carried out essentially as previously described [18,52,53] in 20 mM Tris pH7.5, 150 mM NaCl (TBS) containing 5 mM 2-mercaptoethanol. PI kinase assays contained 2 mM MgCl_2_, 2 mM MnCl_2_, 0.2 mM ATP, 5–10 μCi ^32^P]γATP, 500 μg/ml of phosphatidylinositol (PI) and 250 μg/ml of phosphatidylserine (PS). Extracted phospholipids were separated by thin layer chromatography in 65% 1-propanol, 0.7 M acetic acid, 50 mM phosphoric acid, exposed to a phosphor screen (Molecular Dynamics) and analysed using ImageQuant software (GE Healthcare). Michaelis-Menten kinetics for phosphorylation of PI and PI-(4,5)-P_2_ were calculated from initial reaction rates in assays in which the concentration of ATP was varied between 0 and 125 μM. Reactions were stopped after 20 min using 1 M HCl.

### Protein kinase assays

Protein kinase assays were carried out in TBS containing 2 mM MgCl_2_, 2 mM MnCl_2_, 0.2 mM ATP and 5–10 μCi [^32^P]γATP. Phosphorylated proteins were separated from free [^32^P]γATP by SDS-PAGE as described above. Gels were fixed and stained with Coomassie Blue, then dried, exposed to a phosphor screen and analysed as described above.

### Phosphatase treatment

500 ng recombinant PI3K was mixed with 2–10 U recombinant Calf Intestinal Alkaline Phosphatase, 0.5-2 U Antarctic Phosphatase or 8–40 U Lambda (λ) Protein Phosphatase (NEB) and assayed for PI kinase activity or protein kinase activity as described above.

### In vitro phosphorylation of recombinant p110αEE/p85α

25 μg purified wild type or mutant p110αEE/p85α complex was incubated with or without 1 mM ATP in the presence of 20 mM Tris pH 7.5, 150 mM NaCl, 5 mM 2-mercaptoethanol, 2 mM MgCl_2_ and 2 mM MnCl_2_ at room temperature for 16 hr. Phosphorylated or mock phosphorylated PI3Kα was buffer exchanged into TBS containing 10 mM 2-mercaptoethanol using Sephadex G-25 (GE Life Sciences) and concentrated using centrifugal filters (Amicon Ultra15 10,000 NMWL, Millipore). 250 ng of phosphorylated or mock phosphorylated (control) PI3Kα was assayed for PI kinase activity or protein kinase activity as described above.

Phosphorylation sites on p110αEE/p85α were mapped by digesting 2 μg aliquots of phosphorylated or mock phosphorylated p110αEE/p85α with 0.1 μg trypsin (Worthington) then purifying phosphopeptides using Fe^3+^ immobilised metal affinity chromatography (IMAC) as described [54]. Phosphopeptides were identified by peptide mass fingerprinting using a QSTAR oMALDI-QqTOF (Applied Biosystems/PE Sciex).

## Competing interests

The authors declare that they have no competing interests.

## Authors’ contributions

MJL designed the experiments; MJL, MS and NLC performed the experiments; MJL, MS, RBP and WAP analysed the data; MJL, WAP and RBP interpreted the data; MJL, WAP and RBP wrote the manuscript; MJL, WAP, RBP and CAM revised the manuscript; WAP and CAM gave final approval of the version to be published. All authors read and approved the final manuscript.

## References

[B1] Vanhaesebroeck B Stephens L Hawkins P PI3K signalling: the path to discovery and understanding Nat Rev Mol Cell Biol 2012 13 3 195 203 10.1038/nrm3290 22358332

[B2] Lemmon MA Membrane recognition by phospholipid-binding domains Nat Rev Mol Cell Biol 2008 9 2 99 111 10.1038/nrm2328 18216767

[B3] Cantley LC The phosphoinositide 3-kinase pathway Science 2002 296 5573 1655 1657 10.1126/science.296.5573.1655 12040186

[B4] Samuels Y Wang Z Bardelli A Silliman N Ptak J Szabo S Yan H Gazdar A Powell SM Riggins GJ Willson JK Markowitz S Kinzler KW Vogelstein B Velculescu VE High frequency of mutations of the PIK3CA gene in human cancers Science 2004 304 5670 554 10.1126/science.1096502 15016963

[B5] Kang S Bader AG Vogt PK Phosphatidylinositol 3-kinase mutations identified in human cancer are oncogenic Proc Natl Acad Sci U S A 2005 102 3 802 807 10.1073/pnas.0408864102 15647370 PMC545580

[B6] Isakoff SJ Engelman JA Irie HY Luo J Brachmann SM Pearline RV Cantley LC Brugge JS Breast cancer-associated PIK3CA mutations are oncogenic in mammary epithelial cells Cancer Res 2005 65 23 10992 11000 10.1158/0008-5472.CAN-05-2612 16322248

[B7] Bader AG Kang S Vogt PK Cancer-specific mutations in PIK3CA are oncogenic in vivo Proc Natl Acad Sci U S A 2006 103 5 1475 1479 10.1073/pnas.0510857103 16432179 PMC1360603

[B8] Bachman KE Argani P Samuels Y Silliman N Ptak J Szabo S Konishi H Karakas B Blair BG Lin C Peters BA Velculescu VE Park BH The PIK3CA gene is mutated with high frequency in human breast cancers Cancer Biol Ther 2004 3 8 772 775 10.4161/cbt.3.8.994 15254419

[B9] Campbell IG Russell SE Choong DY Montgomery KG Ciavarella ML Hooi CS Cristiano BE Pearson RB Phillips WA Mutation of the PIK3CA gene in ovarian and breast cancer Cancer Res 2004 64 21 7678 7681 10.1158/0008-5472.CAN-04-2933 15520168

[B10] Velho S Oliveira C Ferreira A Ferreira AC Suriano G Schwartz S Jr Duval A Carneiro F Machado JC Hamelin R Seruca R The prevalence of PIK3CA mutations in gastric and colon cancer Eur J Cancer 2005 41 11 1649 1654 10.1016/j.ejca.2005.04.022 15994075

[B11] Miyaki M Iijima T Yamaguchi T Takahashi K Matsumoto H Yasutome M Funata N Mori T Mutations of the PIK3CA gene in hereditary colorectal cancers Int J Cancer 2007 121 7 1627 1630 10.1002/ijc.22829 17546593

[B12] Liu P Cheng H Roberts TM Zhao JJ Targeting the phosphoinositide 3-kinase pathway in cancer Nat Rev Drug Discov 2009 8 8 627 644 10.1038/nrd2926 19644473 PMC3142564

[B13] Ikenoue T Kanai F Hikiba Y Obata T Tanaka Y Imamura J Ohta M Jazag A Guleng B Tateishi K Asaoka Y Matsumura M Kawabe T Omata M Functional analysis of PIK3CA gene mutations in human colorectal cancer Cancer Res 2005 65 11 4562 4567 10.1158/0008-5472.CAN-04-4114 15930273

[B14] Zhao JJ Liu Z Wang L Shin E Loda MF Roberts TM The oncogenic properties of mutant p110alpha and p110beta phosphatidylinositol 3-kinases in human mammary epithelial cells Proc Natl Acad Sci U S A 2005 102 51 18443 18448 10.1073/pnas.0508988102 16339315 PMC1317954

[B15] Chaussade C Cho K Mawson C Rewcastle GW Shepherd PR Functional differences between two classes of oncogenic mutation in the PIK3CA gene Biochem Biophys Res Commun 2009 381 4 577 581 10.1016/j.bbrc.2009.02.081 19233141

[B16] Foukas LC Beeton CA Jensen J Phillips WA Shepherd PR Regulation of phosphoinositide 3-kinase by its intrinsic serine kinase activity in vivo Mol Cell Biol 2004 24 3 966 975 10.1128/MCB.24.3.966-975.2004 14729945 PMC321424

[B17] Dhand R Hiles I Panayotou G Roche S Fry MJ Gout I Totty NF Truong O Vicendo P Yonezawa K PI 3-kinase is a dual specificity enzyme: autoregulation by an intrinsic protein-serine kinase activity EMBO J 1994 13 3 522 533 8313897 10.1002/j.1460-2075.1994.tb06290.x PMC394841

[B18] Layton MJ Harpur AG Panayotou G Bastiaens PI Waterfield MD Binding of a diphosphotyrosine-containing peptide that mimics activated platelet-derived growth factor receptor beta induces oligomerization of phosphatidylinositol 3-kinase J Biol Chem 1998 273 50 33379 33385 10.1074/jbc.273.50.33379 9837914

[B19] Carson JD Van Aller G Lehr R Sinnamon RH Kirkpatrick RB Auger KR Dhanak D Copeland RA Gontarek RR Tummino PJ Luo L Effects of oncogenic p110alpha subunit mutations on the lipid kinase activity of phosphoinositide 3-kinase Biochem J 2008 409 2 519 524 10.1042/BJ20070681 17877460

[B20] Carpenter CL Auger KR Duckworth BC Hou WM Schaffhausen B Cantley LC A tightly associated serine/threonine protein kinase regulates phosphoinositide 3-kinase activity Mol Cell Biol 1993 13 3 1657 1665 8382773 10.1128/mcb.13.3.1657-1665.1993 PMC359478

[B21] Layton MJ Church NL Faux MC Ji H Goode RJ Kapp EA Burgess AW Simpson RJ Solubilisation of the armadillo-repeat protein beta-catenin using a zwitterionic detergent allows resolution of phosphorylated forms by 2DE Electrophoresis 2012 33 12 1804 1813 10.1002/elps.201100671 22740469

[B22] Lam K Carpenter CL Ruderman NB Friel JC Kelly KL The phosphatidylinositol 3-kinase serine kinase phosphorylates IRS-1. Stimulation by insulin and inhibition by wortmannin J Biol Chem 1994 269 32 20648 20652 8051164

[B23] Foukas LC Shepherd PR eIF4E Binding protein 1 and H-Ras are novel substrates for the protein kinase activity of class-I phosphoinositide 3-kinase Biochem Biophys Res Commun 2004 319 2 541 549 10.1016/j.bbrc.2004.04.191 15178440

[B24] Beeton CA Chance EM Foukas LC Shepherd PR Comparison of the kinetic properties of the lipid- and protein-kinase activities of the p110alpha and p110beta catalytic subunits of class-Ia phosphoinositide 3-kinases Biochem J 2000 350 Pt 2 353 359 10947948 PMC1221261

[B25] Ruiz-Larrea F Vicendo P Yaish P End P Panayotou G Fry MJ Morgan SJ Thompson A Parker PJ Waterfield MD Characterization of the bovine brain cytosolic phosphatidylinositol 3-kinase complex Biochem J 1993 290 Pt 2 609 616 8383968 10.1042/bj2900609 PMC1132318

[B26] Michell RH Harwood JL Coleman R Hawthorne JN Characteristics of rat liver phosphatidylinositol kinase and its presence in the plasma membrane Biochim Biophys Acta 1967 144 3 649 658 10.1016/0005-2760(67)90053-7 4294903

[B27] Van Aller GS Carson JD Fernandes C Lehr R Sinnamon RH Kirkpatrick RB Tummino PJ Luo L Characterization of PI3K class IA isoforms with regulatory subunit p55alpha using a scintillation proximity assay Anal Biochem 2008 383 2 311 315 10.1016/j.ab.2008.08.037 18814837

[B28] Stephens L Jackson T Hawkins PT Synthesis of phosphatidylinositol 3,4,5-trisphosphate in permeabilized neutrophils regulated by receptors and G-proteins J Biol Chem 1993 268 23 17162 17172 8394332

[B29] van der Kaay J Batty IH Cross DA Watt PW Downes CP A novel, rapid, and highly sensitive mass assay for phosphatidylinositol 3,4,5-trisphosphate (PtdIns(3,4,5)P3) and its application to measure insulin-stimulated PtdIns(3,4,5)P3 production in rat skeletal muscle in vivo J Biol Chem 1997 272 9 5477 5481 10.1074/jbc.272.9.5477 9038150

[B30] Sasaki T Takasuga S Sasaki J Kofuji S Eguchi S Yamazaki M Suzuki A Mammalian phosphoinositide kinases and phosphatases Prog Lipid Res 2009 48 6 307 343 10.1016/j.plipres.2009.06.001 19580826

[B31] Dyson JM Fedele CG Davies EM Becanovic J Mitchell CA Phosphoinositide phosphatases: just as important as the kinases Sub-cellular biochemistry 2012 58 215 279 10.1007/978-94-007-3012-0_7 22403078

[B32] Yu J Zhang Y McIlroy J Rordorf-Nikolic T Orr GA Backer JM Regulation of the p85/p110 phosphatidylinositol 3’-kinase: stabilization and inhibition of the p110alpha catalytic subunit by the p85 regulatory subunit Mol Cell Biol 1998 18 3 1379 1387 9488453 10.1128/mcb.18.3.1379 PMC108851

[B33] Yu J Wjasow C Backer JM Regulation of the p85/p110alpha phosphatidylinositol 3’-kinase. Distinct roles for the n-terminal and c-terminal SH2 domains J Biol Chem 273 273 46 30199 30203 9804776 10.1074/jbc.273.46.30199

[B34] Backer JM Myers MG Jr Shoelson SE Chin DJ Sun XJ Miralpeix M Hu P Margolis B Skolnik EY Schlessinger J Phosphatidylinositol 3’-kinase is activated by association with IRS-1 during insulin stimulation EMBO J 1992 11 9 3469 3479 1380456 10.1002/j.1460-2075.1992.tb05426.x PMC556882

[B35] Rordorf-Nikolic T Van Horn DJ Chen D White MF Backer JM Regulation of phosphatidylinositol 3’-kinase by tyrosyl phosphoproteins. Full activation requires occupancy of both SH2 domains in the 85-kDa regulatory subunit J Biol Chem 1995 270 8 3662 3666 10.1074/jbc.270.8.3662 7876105

[B36] Carpenter CL Auger KR Chanudhuri M Yoakim M Schaffhausen B Shoelson S Cantley LC Phosphoinositide 3-kinase is activated by phosphopeptides that bind to the SH2 domains of the 85-kDa subunit J Biol Chem 1993 268 13 9478 9483 7683653

[B37] Miled N Yan Y Hon WC Perisic O Zvelebil M Inbar Y Schneidman-Duhovny D Wolfson HJ Backer JM Williams RL Mechanism of two classes of cancer mutations in the phosphoinositide 3-kinase catalytic subunit Science 2007 317 5835 239 242 10.1126/science.1135394 17626883

[B38] Mandelker D Gabelli SB Schmidt-Kittler O Zhu J Cheong I Huang CH Kinzler KW Vogelstein B Amzel LM A frequent kinase domain mutation that changes the interaction between PI3Kalpha and the membrane Proc Natl Acad Sci U S A 2009 106 40 16996 17001 10.1073/pnas.0908444106 19805105 PMC2761334

[B39] Rodriguez-Viciana P Warne PH Dhand R Vanhaesebroeck B Gout I Fry MJ Waterfield MD Downward J Phosphatidylinositol-3-OH kinase as a direct target of Ras Nature 1994 370 6490 527 532 10.1038/370527a0 8052307

[B40] Pleiman CM Hertz WM Cambier JC Activation of phosphatidylinositol-3’ kinase by Src-family kinase SH3 binding to the p85 subunit Science 1994 263 5153 1609 1612 10.1126/science.8128248 8128248

[B41] Tolias KF Cantley LC Carpenter CL Rho family GTPases bind to phosphoinositide kinases J Biol Chem 1995 270 30 17656 17659 10.1074/jbc.270.30.17656 7629060

[B42] Klippel A Reinhard C Kavanaugh WM Apell G Escobedo MA Williams LT Membrane localization of phosphatidylinositol 3-kinase is sufficient to activate multiple signal-transducing kinase pathways Mol Cell Biol 1996 16 8 4117 4127 8754810 10.1128/mcb.16.8.4117 PMC231408

[B43] Rodriguez-Viciana P Warne PH Vanhaesebroeck B Waterfield MD Downward J Activation of phosphoinositide 3-kinase by interaction with Ras and by point mutation EMBO J 1996 15 10 2442 2451 8665852 PMC450176

[B44] von Willebrand M Williams S Saxena M Gilman J Tailor P Jascur T Amarante-Mendes GP Green DR Mustelin T Modification of phosphatidylinositol 3-kinase SH2 domain binding properties by Abl- or Lck-mediated tyrosine phosphorylation at Tyr-688 J Biol Chem 1998 273 7 3994 4000 10.1074/jbc.273.7.3994 9461588

[B45] Lee JY Chiu YH Asara J Cantley LC Inhibition of PI3K binding to activators by serine phosphorylation of PI3K regulatory subunit p85alpha Src homology-2 domains Proc Natl Acad Sci U S A 2011 108 34 14157 14162 10.1073/pnas.1107747108 21825134 PMC3161555

[B46] Huang CH Mandelker D Schmidt-Kittler O Samuels Y Velculescu VE Kinzler KW Vogelstein B Gabelli SB Amzel LM The structure of a human p110alpha/p85alpha complex elucidates the effects of oncogenic PI3Kalpha mutations Science 2007 318 5857 1744 1748 10.1126/science.1150799 18079394

[B47] Vadas O Burke JE Zhang X Berndt A Williams RL Structural basis for activation and inhibition of class I phosphoinositide 3-kinases Sci Signal 2011 4 195 re2 10.1126/scisignal.2002165 22009150

[B48] Porfiri E Evans T Chardin P Hancock JF Prenylation of Ras proteins is required for efficient hSOS1-promoted guanine nucleotide exchange J Biol Chem 1994 269 36 22672 22677 8077219

[B49] O’Reilly DR Miller LK Luckow VA Baculovirus expression vectors: a laboratory manual 1994 Oxford University Press, Oxford

[B50] Catimel B Layton M Church N Ross J Condron M Faux M Simpson RJ Burgess AW Nice EC In situ phosphorylation of immobilized receptors on biosensor surfaces: application to E-cadherin/beta-catenin interactions Anal Biochem 2006 357 2 277 288 10.1016/j.ab.2006.07.034 16945320

[B51] Wong C Sridhara S Bardwell JC Jakob U Heating greatly speeds coomassie blue staining and destaining Biotechniques 2000 28 3 426 428 430, 432 10723553 10.2144/00283bm07

[B52] Whitman M Kaplan DR Schaffhausen B Cantley L Roberts TM Association of phosphatidylinositol kinase activity with polyoma middle-T competent for transformation Nature 1985 315 6016 239 242 10.1038/315239a0 2987699

[B53] Arcaro A Volinia S Zvelebil MJ Stein R Watton SJ Layton MJ Gout I Ahmadi K Downward J Waterfield MD Human phosphoinositide 3-kinase C2beta, the role of calcium and the C2 domain in enzyme activity J Biol Chem 1998 273 49 33082 33090 10.1074/jbc.273.49.33082 9830063

[B54] Stensballe A Steen H Jensen ON Simpson RJ Proteomic methods for phosphorylation site mapping Proteins and proteomics: a laboratory manual 2003 Cold Spring Harbor Laboratory Press, Cold Spring Harbor, NY 926 xiii

